# Individuals with Higher Levels of Physical Activity after Stroke Show Comparable Patterns of Myelin to Healthy Older Adults

**DOI:** 10.1177/15459683221100497

**Published:** 2022-05-09

**Authors:** Brian Greeley, Cristina Rubino, Ronan Denyer, Briana Chau, Beverley Larssen, Bimal Lakhani, Lara Boyd

**Affiliations:** 1 Department of Physical Therapy, 8166 University of British Columbia, Vancouver, BC, Canada; 2 Graduate Program in Rehabilitation Sciences, 8166 University of British Columbia, Vancouver, BC, Canada; 3 Graduate Program in Neuroscience, 8166 University of British Columbia, Vancouver, BC, Canada

**Keywords:** Chronic stroke, myelin water fraction, physical activity, asymmetry ratios, older adults

## Abstract

**Background:**

Myelin asymmetry ratios (MARs) relate and contribute to motor impairment and function after stroke. Physical activity (PA) may induce myelin plasticity, potentially mitigating hemispheric myelin asymmetries that can occur after a stroke.

**Objective:**

The aim of this study was to determine whether individuals with higher levels of PA showed lower MAR compared to individuals with lower levels of PA.

**Methods:**

Myelin water fraction was obtained from 5 bilateral motor regions in 22 individuals with chronic stroke and 26 healthy older adults. Activity levels were quantified with wrist accelerometers worn for a period of 72 hours (3 days). Higher and lower PA levels were defined by a cluster analysis within each group.

**Results:**

MAR was similar regardless of PA level within the older adult group. Compared to the higher PA stroke group, lower PA stroke participants displayed greater MAR. There was no difference in MAR between the stroke and older adult higher PA groups. Within the lower PA groups, individuals with stroke showed greater MAR compared to the older adults. Arm impairment, lesion volume, age, time since stroke, and preferential arm use were not different between the PA stroke groups, suggesting that motor impairment severity and extent of brain damage did not drive differences in PA.

**Conclusion:**

Individuals who have had a stroke and are also physically active display lower MAR (i.e., similar myelin in both hemispheres) in motor regions. High levels of PA may be neuroprotective and mitigate myelin asymmetries once a neurological insult, such as a stroke, occurs. Alternately, it is possible that promoting high levels of PA after a stroke may reduce myelin asymmetries.

## Introduction

Due to a reduction in mortality rates, there are an increasing number of individuals living with long-term disabilities post-stroke. Consequently, people with stroke have the highest need for rehabilitation among neurological disorders worldwide.^1^ Identifying effective interventions that optimize recovery of motor function represents an important challenge to improve quality of life after stroke.

Inducing myelin plasticity has become a viable therapeutic target for improving recovery after stroke.^2,3^ White matter plays a crucial role in the formation and function of neural circuits^4,5^ and undergoes use-dependent plasticity in young^6,7^ and older^8^ adults. However, following a stroke, there is considerable loss of myelin in both the contra- and ipsilesional hemispheres,^9-11^ which contributes to sensorimotor deficits.^2,9,12^ Specifically, myelin asymmetry ratios (MARs), calculated as a ratio of contralesional to ipsilesional myelin water fraction, in the posterior limb of the internal capsule are greater (e.g., >1 and therefore less symmetrical) in individuals who have had a stroke compared with older adults.^11^ Additionally, there is a negative relationship between MAR in the precentral gyrus^9^ and corticospinal tract^13,14^ and upper-extremity motor impairment. Approaches that target and reduce MAR may also improve function after stroke.

Physical activity (PA) induces white matter plasticity. In animal models, exercise increased myelin debris removal and enhanced remyelination in chronic cerebral hypoperfusion rats,^15^ and increased the rate of remyelination after a demyelinating injury.^16^ In older adults, there is a positive relationship between white matter structure in the fornix, temporal, and frontal brain regions and amount of PA.^17,18^ Further, aerobic exercise and resistance training increases white matter volume in the prefrontal cortex^19^ and decreases white matter lesion volume,^20^ respectively. Taken together, PA appears to be a promising, cost-effective approach to promote white matter plasticity in older adults. An open question, however, is whether individuals who are more physically active have more symmetrical MAR (i.e., values close to 1). Yet, PA is often obtained through self-report questionnaires, which are subjective and may not accurately reflect real-world activity.^21^

The current study investigated MAR (contralesional/ipsilesional or dominant/non-dominant hemispheres) from five motor regions of interest (ROIs) in low and high physically active individuals with chronic stroke (>6 months) and older adults. Physical activity levels were obtained using accelerometers which participants wore for 72 consecutive hours (3 days). We hypothesized that: (1) individuals with stroke would display greater MAR (i.e., >1) relative to older adults, and (2) individuals in the lower PA stroke group would display greater MAR in motor ROIs relative to individuals in the higher PA stroke group as well as the older adult group.

## Methods

### Participants

Thirty individuals with chronic stroke (>6 months)^22^ were recruited to participate in the study along with 27 older healthy controls. All participants were between the ages of 40 and 85 and had no contraindications to magnetic resonance imaging (MRI). Participants were excluded if they had a history of head trauma, seizures, psychiatric diagnosis, or a neurodegenerative or neurological disorder other than stroke. Informed consent was obtained prior to the administration of any experimental protocol. The Clinical Research Ethics Board at the University of British Columbia approved all protocols.

### Experimental Design

Participants underwent MRI, and individuals with stroke completed the upper-extremity portion of the Fugl-Meyer (UE-FM)^23^ (see *Motor assessments* below). Participants wore accelerometers placed on their wrists for 3 consecutive days (72 hours). It should be noted that the data presented here are from the baseline portion of a larger, 10-day upper-extremity study. To date, we have not published any of the accelerometer or myelin water fraction data.

### MRI Acquisition

MRI data were acquired on a 3.0 T Philips Achieva whole body MRI (Philips Healthcare, Best, NL). Scans used an eight-channel SENSE head coil and parallel imaging and included: (1) 3D T_1_ turbo field echo (TE/TR = 3.6/7.4 ms, flip angle θ = 6°, FOV = 256 × 256 mm^2^, 160 slices, 1 mm slice thickness, scan time = 3.2 min); and (2) whole-cerebrum 32-echo 3D Gradient and Spin Echo (GRASE) for T_2_ measurement (TE/TR = 10/1000 ms, echo times = 10-320 ms with 10 ms spacing, 20 slices acquired at 5 mm slice thickness, 40 slices reconstructed at 2.5 mm slice thickness (zero filled interpolation), slice oversampling factor = 1.3 (26 slices were actually acquired but only the central 20 were reconstructed), in-plane voxel size = 1 × 1 mm^2^, SENSE = 2, 232 × 192 matrix, receiver bandwidth = 188 kHz, axial orientation, acquisition time = 14.4 min).^24^ T_1_-weighted and GRASE sequences were obtained in the same session.

### MRI Pre-Processing

Using the 32-echo GRASE data, voxel-wise T_2_ distributions were calculated using a non-negative least-squares algorithm with the Extended Phase Graph algorithm and flip angle optimization (in-house software developed at the University of British Columbia using MATLAB R2010b, The MathWorks, Inc.; analysis code can be requested here: https://mriresearchWi.med.ubc.ca/news-projects/myelin-water-fraction/).^24,25^ MWF was defined as the sum of the amplitudes within a short T_2_ signal (15–40 ms) divided by the sum of the amplitudes for the total T_2_ distribution and voxel-wise MWF maps were created for each participant.

### Myelin Water Fraction Region of Interest Analysis

The first echo of the GRASE scan was linearly registered to their respective T_1_ scans using FSL FLIRT.^26^ T_1_ scans were then non-linearly registered (using FSL FNIRT) to 1 mm MNI space. Transformation matrices were inverted to create a warping field of MNI to GRASE space. This warp was used to transform John Hopkins University International Consortium of Brain Mapping (ICBM) DTI-81 white matter ROIs^27^ to GRASE space. Mean MWF for each ROI in GRASE space was extracted using FSL STATS. Stroke lesion masks (created manually in T_1_ space) were used to assist with registration and to subtract the stroke lesion from ROIs before extracting MWF. Given their importance in motor recovery and function after stroke,^28-34^ the following motor ROIs were chosen a priori: anterior limb of internal capsule (ALIC), cerebral peduncle (CP), posterior corona radiata (PCR), posterior limb of the internal capsule (PLIC), and superior corona radiata (SCR). MWF asymmetry ratio was calculated for each ROI using the following equation

$$\text{MAR}=\frac{\text{MWF contralesional or dominant hemisphere}}{\text{MWF ipsilesional or non}-\text{dominant hemisphere}}$$

Greater values correspond to a greater asymmetry skewed toward the contralesional/dominant hemisphere for a given ROI.

### Activity Monitoring

Actical accelerometers (Phillips, Amsterdam, Netherlands) were used to measure participants’ PA level. Accelerometers were placed on the participants’ left and right wrists,^35-39^ and worn for 72 consecutive hours and were told to go about their normal daily activities. The accelerometers are lightweight, small (28 × 27 × 10 mm^3^), and waterproof. Arm activity was sampled at 32 Hz and binned into 15 second epochs. Movement occurring during each epoch was converted from mechanical motion into an electrical signal using the Actical Software package and stored as the intensity of the activity performed during the interval of time specified. Activity counts collected were averaged to determine total activity (affected or unaffected for stroke, non-dominant or dominant for older adults). Previously reported findings have established high test-retest reliability (*r* > .86) of accelerometers for measurement of arm activity in stroke, with 72 consecutive hours (3 days) been validated as an index of arm activity during normal daily activities in chronic stroke.^40,41^

### Motor Assessments

FM-UE was administered by trained physiotherapists and was used to quantify upper-extremity impairment. The upper-extremity portion of the Fugl-Meyer is out of a total of 66 points.^23^

### Statistical Analysis

We performed cluster analyses to categorize participants into either higher or lower PA levels. One advantage of a this analysis is it allows the classification of similar observations into clusters with high internal homogeneity and external heterogeneity,^42^ yielding more informative and consistent findings than median split methods.^43,44^ The variables entered in the cluster analyses were averaged activity values which were quantified from the accelerometers. The variables were as follows: the amount of wrist steps, percentage of time spent in sedentary activity, percentage of time spent in light activity, percentage of time spent in moderate activity, and percentage of time spent in vigorous activity. We allowed for a maximum of 10 iterations, with a convergence criterion of 0.

Next, we performed a mixed repeated measures ANOVA. We performed a 2 (Group: older adults and stroke) by 2 (Activity Level: high and low) by 5 (ROI: ALIC, CP, PCR, PLIC, and SCR) by repeated measures ANOVA, with Group and Activity Level as between-subjects variables and ROI as a within-subject variable. Post hoc pairwise comparisons were Sidak corrected.

To better understand what was driving group differences between the higher and lower PA stroke groups, we performed multiple one-way ANOVAs comparing FM-UE scores, lesion volume, age, and time since stroke between the 2 physically active stroke groups. We also performed a series of 2 Arm (Arm: contralesional and ipsilesional) by 2 (Activity Level: high and low) repeated measures ANOVAs comparing each PA metric used in the cluster analysis (steps, percentage in sedentary, light, and moderate activity).

Finally, to understand brain structure and arm function relationships, we also conducted exploratory analyses using multiple Spearman bivariate correlations between MAR and asymmetry ratios of wrist steps (e.g., contralesional or dominant wrist/ipsilesional or non-dominant wrist) by pooling the data from all participants in the stroke and older adult groups. Because previous work has demonstrated a relationship between myelin and age,^9^ correlation analyses of MAR within each ROI and age were first conducted and ROIs that were significantly correlated with age were removed from further analyses to avoid spurious correlations. Due to the exploratory nature of this analysis, we did not correct for multiple comparisons.

All variables were tested for normality using the Shapiro–Wilk test (P < .01).^45^ If normality was violated, variables were natural log transformed or outliers were removed. All statistical analyses were carried out using SPSS version 23 (IBM Corp).

## Results

We could not extract MWF values from 5 stroke participants due to registration issues in FSL (involving registration issues of T_1_ to MNI or MNI to GRASE space). Three additional participants were excluded (2 stroke and 1 older adult) due to incomplete wrist accelerometry data and 1 participant was excluded from the lower PA stroke group due to an extreme PLIC MAR (>3 SD). Therefore, the statistical analyses included 22 stroke participants and 26 older adults. All participant demographic characteristics are presented in Table 1.

**Table 1.** Demographic data of participants. Hem = hemisphere; TSS = time since stroke in months.

	Participant ID	Sex	Age	Activity level	Lesion volume (Voxel)	Stroke Hem	TSS	Fugl-Meyer score
**Stroke**	1	F	57	Low	1910	L	162	32
	2	F	51	Low	8792	R	47	29
	3	M	58	Low	31 282	L	96	59
	4	M	72	High	261	R	49	50
	5	M	71	High	208	R	117	59
	6	M	77	High	52	L	32	58
	7	F	70	Low	220	R	76	64
	8	M	60	Low	34 024	R	188	31
	9	M	73	Low	237	L	60	54
	10	M	69	Low	268	L	66	25
	11	F	37	High	1863	L	84	18
	12	M	79	High	480	R	27	54
	13	M	62	High	324	R	8	59
	14	M	80	Low	738	R	40	62
	15	F	78	High	74	R	28	64
	16	M	75	Low	1051	L	41	65
	17	M	59	Low	29 095	L	61	33
	18	M	61	Low	1198	L	62	28
	19	F	67	Low	44	L	94	52
	20	F	74	Low	1588	R	19	39
	21	M	51	Low	590	R	14	23
	22	M	62	High	1254	L	6	62
	Mean (SD)	7 F/15 M	65.6 (10.9)	8 H/14 L	5252.4 (10 841.6)	11 L/11 R	54.5 (44.7)	46.4 (16.0)
								
Older adults	1	M	67	High				
	2	M	72	Low				
	3	F	63	High				
	4	M	81	Low				
	5	F	58	High				
	6	F	74	High				
	7	F	62	High				
	8	F	62	High				
	9	F	61	Low				
	10	M	63	High				
	11	M	51	High				
	12	F	73	Low				
	13	F	67	High				
	14	M	75	Low				
	15	M	71	Low				
	16	F	65	High				
	17	F	77	Low				
	18	F	63	Low				
	19	M	61	Low				
	20	F	58	Low				
	21	M	68	High				
	22	M	58	Low				
	23	F	47	High				
	24	F	50	High				
	25	F	58	Low				
	26	F	73	High				
	Mean (SD)	16 F/10 M	64.5 (8.5)	14 H/12 L				

### Cluster Analysis

Cluster analysis revealed 14 older adults with higher and 12 with lower PA levels, respectively. In contrast, there were 8 individuals in the stroke group classified as having higher PA levels and 14 individuals in the stroke group classified as having lower PA levels (Table 2).

**Table 2.** Wrist accelerometry data averaged across arms across 3 days. Values are presented as mean (SD).

	Older adults		Stroke	
	High (n = 14)	Low (n = 12)	High (n = 8)	Low (n = 14)
Steps number	19 839.1 (5910.6)	9959.0 (2549.7)	16 225.7 (2893.8)	7000.2 (2332.9)
Sedentary	49.6% (7.3)	65.6% (7.2)	56.5% (4.4)	70.7% (7.4)
Light	21.3% (4.4)	18.4% (4.3)	21.2% (2.5)	18.7% (5.1)
Moderate	28.5% (6.4)	16.0% (4.5)	22.0% (3.1)	10.6% (3.5)
Vigorous	.6% (.7)	.0% (.1)	.3% (.6)	.0% (.0)

### Myelin Water Fraction Asymmetry Ratios

Ipsi-/non-dominant and contralesional/dominant myelin water fraction values can be viewed in Supplementary Table 1. The mixed repeated measures ANOVA revealed a main effect of Group (*F*(1,44) = 18.080, P < .001, η_p_^2^ = .29) with greater MAR for the stroke group (M = 1.152, SE ± .028) relative to the older adults (M = .991, SE ± .025; Supplementary Figure 1). There was also a main effect of Physical Activity Level (*F*(1,44) = 6.203, P = .017, η_p_^2^ = .12) with individuals in the low PA group showing greater MAR (M = 1.119, SE ± .025) relative to the high PA group (M = 1.024, SE ± .033; Supplementary Figure 2).

**Figure 1.**
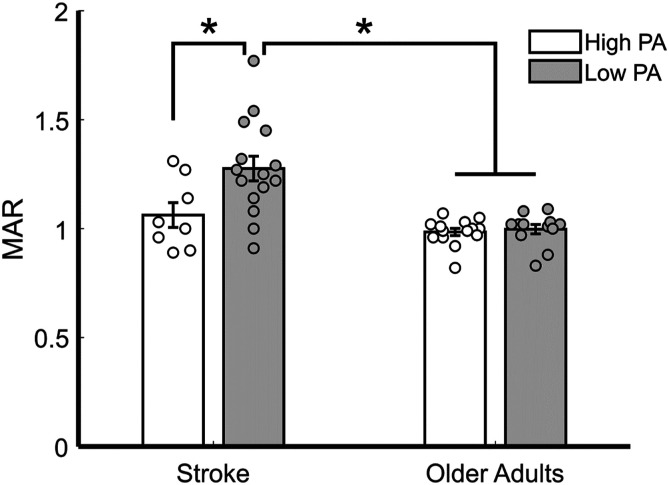
Mean myelin asymmetry ratios (MARs) for physical activity (PA) group in individuals who have had a stroke and older adults. There was a significant group by PA level interaction (P = .031). The low PA stroke group had greater MAR compared to the high PA stroke group (P = .032). There was no difference between the PA groups within older adults (P = .691). Within the high PA group there was no difference between the stroke and older adult group (P = .122), whereas within the low PA group, stroke had an overall greater MAR compared to older adults (P < .001). Error bars represent standard error. Circles represent individual datapoints.

There was also a Group by Physical Activity Level interaction (*F*(1,44) = 4.854, P = .033, η_p_^2^ = .10) driven by a lack of difference between older adults in the low PA level (M = .997, SE ± .043) and the high PA level group (P = .691; M = .986, SE ± .039), while individuals who had a stroke in the low PA group displayed significantly greater MAR (M = 1.241, SE ± .034) compared to individuals who had a stroke in the high PA group (P = .032; M = 1.063, SE ± .045; Figure 1). Importantly, within the high PA group, there was no significant difference between individuals with stroke and older adults (P = .122), whereas within the low physical active group, individuals with stroke displayed a significantly greater MAR compared to the older adults (P <.001; Figure 1).

Finally, to understand how time since stroke and motor impairment as measured by the Fugl-Meyer impacts MAR, we performed 3 separate mixed repeated measures ANOVAs limited to the stroke group. We used the same 5 ROIs as the within-subjects factor and Physical Activity Level (high and low) as the between-subject factor. In each of the 3 analyses, we used time since stroke, FM score, or time since stroke and FM score as a covariate, respectively. We found no main effect of physical activity level emerge when using time since stroke (P = .071), logged FM score (P = .059), or using both time since stroke and logged FM score (P = .097) as covariates. It should be noted that while there is no longer a difference between the 2 stroke groups, our main comparison is between the older adult group and those with high and low PA levels in the stroke group.

### High and Low Physical Activity Stroke Groups

There was no main effect of ROI (*F*(4,176) = .194, P = .941, η_p_^2^ < .01), and no ROI by Group (*F*(4,176) = 2.235, P = .067, η_p_^2^ = .05), ROI by Physical Activity Level (*F*(4,176) = .466, P = .761, η_p_^2^ = .01), and ROI by Group by Physical Activity Level (*F*(4,176) = .400, P = .809, η_p_^2^ = .01) interactions.

There was no difference between logged UE-FM scores (*F*(1,20) = .730, P = .403), logged lesion volume (*F*(1,20) = 3.901, P = .062), age (*F*(1,20) = .221, P = .643), or time since stroke (*F*(1,20) = 2.088, P = .403) between the high and low physically active stroke groups.

To understand which arm (ipsilesional or contralesional) drove the overall difference between the 2 PA level stroke groups, we completed a series of post hoc repeated measures ANOVAs. There was no Arm by Activity Level interactions for number of wrist steps (*F*(1,20) = .312, P = .583), percent time spent in sedentary activity (*F*(1,20) = .696, P = .414), percent time spent in light activity (*F*(1,20) = .035, P = .853), and percent time spent in moderate activity (*F*(1,20) = 1.658, P = .213). Because there were only 4 and 3 non-zero values for percent time spent in vigorous activity for the contralesional and ipsilesional wrist, respectively, we did not complete a test for this metric. Therefore, the high and low physically active groups were using both arms equally.

### Bivariate correlations of MAR and asymmetry of wrist use across all participants (stroke and older adults)

MAR in the CP ROI was significantly related to age, so it was excluded from the exploratory, bivariate analysis. We observed a significant relationship emerge between the wrist steps asymmetry ratios and MAR in the ALIC (*r*_*s*_ = .45, P = .001, n = 48), PCR (*r*_*s*_ = .35, P = .016, n = 48), and SCR (*r*_*s*_ = .31, P = .033, n = 48). MARs in the PLIC were not related to wrist asymmetry ratios (*r*_*s*_ = .09, P = .561, n = 48).

## Discussion

Our data suggest that physical activity levels may affect patterns of myelination after stroke. Stroke damages white matter,^9-11^ which has a negative impact on motor recovery^11^ and contributes to motor impairment.^9^ Here, individuals in the high PA stroke group had comparable MAR to that noted in older adults. In contrast, individuals in the low PA stroke group showed greater MAR relative to older adults. These differences were not related to arm motor impairment, age, lesion volume, time since stroke, or preferential arm use all of which did not differ between the high and low PA stroke groups. This suggests that motor impairment was not a barrier to being more physically active, and that individuals who engaged in low levels of PA have the capacity to be more active. Further, in three ROIs (ALIC, PCR, and SCR), MAR was positively related to wrist use across all participants, with those having greater MAR also displaying greater asymmetry in arm use. Taken together these results suggest that PA serves to mitigate MAR and that maintaining high levels of PA is associated with a normative range of MAR after stroke.

Findings from the current study provide a foundation for future work. Our data raise the question: when (if at all) will engaging in high levels of PA mitigate the negative impact of a stroke on MAR? After stroke, the central nervous system is in a period of heightened plasticity with synaptogenesis,^46^ distal dendritic growth,^47^ and turnover of dendritic spines and vascular axonal remodeling.^48^ Interestingly, exercise enhances the rate of remyelination immediately following a demyelinating insult in mice.^16^ Therefore, it seems reasonable that engaging in high levels of PA can exploit the period of heightened plasticity following stroke which may mitigate the negative impact of stroke on myelin. Additional research is required to better understand the how PA linked to myelination change across phases of stroke recovery.

Our findings also support the idea that engaging in high levels of PA may be neuroprotective. Animal model studies have demonstrated a myriad of plausible mechanisms in which PA is neuroprotective.^49^ It is possible that individuals in the high PA group were also physically active prior to their stroke, which in turn may have mitigated the negative effects of brain damage on MAR. Data from the older adults may also elucidate this neuroprotective process. MAR did not differ between the high and low PA older adult groups, suggesting that while engaging in high levels of PA is neuroprotective, it may be that only once a neurological insult occurs (e.g., advanced aging and stroke) that the benefits of PA are evident. Furthermore, maintenance of high levels of PA after brain injury may serve to sustain this neuroprotective benefit. Longitudinal databases that include MRI and PA measures might be used to test this neuroprotection hypothesis.

White matter projection fibers such as the corona radiata,^34,50^ PLIC,^28,33,34^ ALIC,^33^ and the cerebral peduncles (CP)^31^ play an integral role in upper-extremity motor recovery after stroke. While we did not observe a main effect or interaction involving ROI, the exploratory correlations revealed positive associations between asymmetry in arm use and MAR limited to ALIC, PCR, and SCR across all participants (i.e., stroke and older adults). Unlike the CP and PLIC, which are strictly motoric projection tracts, the ALIC,^51^ SCR, and PCR^52^ have been implicated in both cognitive and motor function. Anatomically, ALIC and the corona radiata carry thalamic and brainstem fibers from prefrontal and parietal cortices.^53^ It may be that projection tracts that subserve cognitive-motor functions may be more susceptible to change and/or influenced by PA compared with strictly motoric projection tracts. For example, in older adults, exercise increased white matter volume in the prefrontal cortex^19^ and in the corona radiata^54^; and individuals that reported higher levels of PA displayed greater white matter in the ALIC.^55^

The lack of a difference between the high and low PA stroke groups in terms of arm motor impairment, number of steps, and percentage of time spent in each activity level for each arm was unexpected. Previous work found that MARs in the precentral gyrus^9^ and the corticospinal tract^13,14^ relate to arm motor impairment in stroke. Based on these previous findings, we expected to observe an arm impairment difference between the low and high PA stroke groups that also showed high and low MARs, respectively. The lack of difference may be due to our methodological approach. We used five ROIs, encompassing various motor regions, whereas previous research has solely focused on the corticospinal tract. It is possible that the addition of other motor regions such as the posterior and superior corona radiata, which have reciprocal connections to other cortical regions such as the primary auditory cortex and the visual cortex,^56^ may account for this difference.

This study had several limitations. Since it was a cross-sectional study, it is currently unknown *when* PA contributes, and is most impactful, to mitigating MAR after stroke. This makes it difficult to form precise recommendations as to when to prescribe exercise. Second, while we followed recommendations and collected physical activity over the course of 72 hours (3 days),^57,58^ a longer duration (e.g., 7 days) would have likely been more accurate. Additionally, the cluster analysis resulted in a smaller sample for the high PA stroke group. While we acknowledge this is a limitation of using a cluster analysis, a median split would have resulted in less informative and inconsistent findings.^43,44^ Future studies should consider recruiting larger samples sizes including more physically active stroke participants to understand whether the same pattern of results is still present in a larger population.

In conclusion, we found that after stroke, individuals who are more physically active have less myelin asymmetry in brain regions known to support movement. This finding was not a result of preferential arm use or differences in motor impairment between people with stroke who engaged in low vs high levels of physical activity. Our data support the beneficial role of physical activity for brain health, especially following stroke.

## Supplemental Material

sj-pdf-1-nnr-10.1177_15459683221100497 - Individuals with Higher Levels of Physical Activity after Stroke Show Comparable Patterns of Myelin to Healthy Older Adults Supplemental material, sj-pdf-1-nnr-10.1177_15459683221100497 for Individuals with Higher Levels of Physical Activity after Stroke Show Comparable Patterns of Myelin to Healthy Older Adults by Brian Greeley, Cristina Rubino, Ronan Denyer, Briana Chau, Beverley Larssen, Bimal Lakhani and Lara Boyd in Neurorehabilitation and Neural Repair
