# Supplementary material for: Individuals with Higher Levels of Physical Activity after Stroke Show Comparable Patterns of Myelin to Healthy Older Adults
Source: Neurorehabil Neural Repair. 2022 May 9;36(6):381–9. doi: 10.1177/15459683221100497 (PMC9127936; doi:10.1177/15459683221100497)
Supplement: sj-pdf-1-nnr-10.1177_15459683221100497 - Individuals with Higher Levels of Physical Activity after Stroke Show Comparable Patterns of Myelin to Healthy Older Adults [file sj-pdf-1-nnr-10.1177_15459683221100497.pdf]

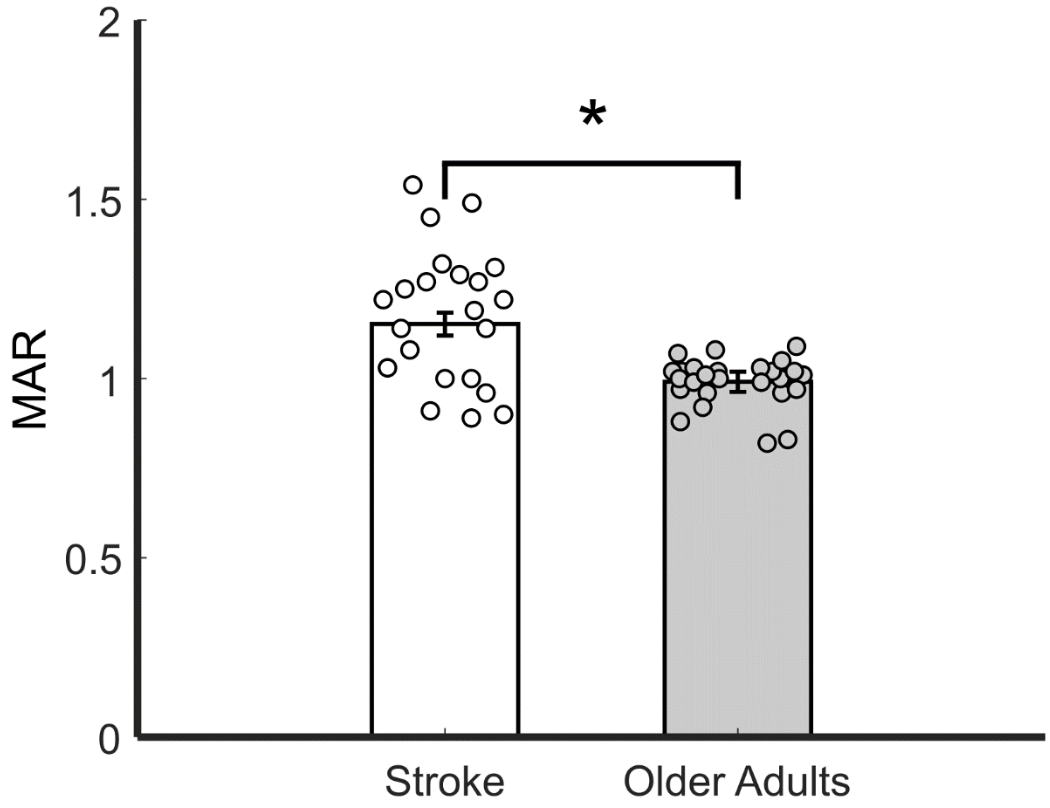

Figure 1. Mean myelin asymmetry ratios (MAR) for stroke (n = 22; white bar) and older adults (n = 26; gray bar). The stroke group had greater MAR all regions of interest relative to older adults ($p < 0.001$). Error bars represent standard error. Circles represent individual datapoints.

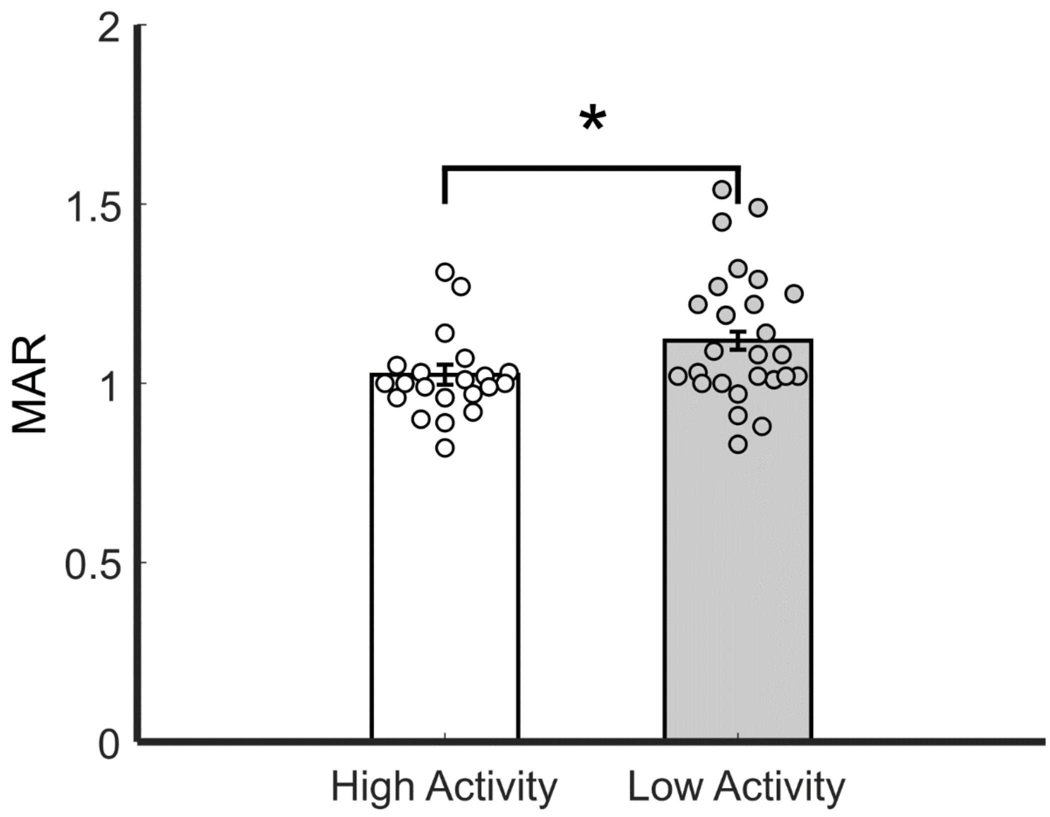

Figure 2. Mean myelin asymmetry ratios (MAR) for high activity individuals (n = 22; white bar) and low activity individuals (n = 26; gray bar). The low activity group had greater MAR across all regions of interest relative to the low activity group (p = .017). Error bars represent standard error. Circles represent individual datapoints.

Table 1. Raw myelin water fraction values for each hemisphere (ipsilesional / non-dominant; contralesional / dominant) for stroke and older adults. ALIC = anterior limb of the internal capsule; Contra = contralesional; CP = cerebral peduncles; D = dominant hemisphere; Ipsi = ipsilesional; ND = non-dominant PCR = posterior corona radiata; PLIC = posterior limb of the internal capsule; SCR = superior corona radiata

		ALIC		CP		PCR		PLIC		SCR	
		Ipsi / ND	Contra / D	Ipsi / ND	Contra / D	Ipsi / ND	Contra / D	Ipsi / ND	Contra / D	Ipsi / ND	Contra / D
**Stroke**	1	0.10	0.13	0.20	0.12	0.12	0.14	0.18	0.18	0.12	0.16
	2	0.08	0.15	0.34	0.09	0.10	0.11	0.14	0.20	0.09	0.13
	3	0.07	0.15	0.34	0.15	0.13	0.12	0.20	0.18	0.13	0.14
	4	0.13	0.15	0.27	0.10	0.12	0.10	0.11	0.09	0.07	0.05
	5	0.07	0.06	0.28	0.10	0.07	0.07	0.15	0.16	0.10	0.09
	6	0.08	0.11	0.20	0.07	0.10	0.10	0.14	0.16	0.06	0.11
	7	0.08	0.10	0.19	0.21	0.10	0.12	0.10	0.19	0.06	0.12
	8	0.11	0.08	0.21	0.25	0.06	0.10	0.11	0.22	0.07	0.15
	9	0.08	0.11	0.22	0.24	0.09	0.13	0.17	0.17	0.11	0.13
	10	0.12	0.12	0.27	0.29	0.11	0.11	0.21	0.14	0.15	0.11
	11	0.07	0.14	0.21	0.31	0.09	0.11	0.09	0.20	0.14	0.15
	12	0.11	0.11	0.42	0.27	0.13	0.13	0.13	0.21	0.14	0.13
	13	0.11	0.07	0.28	0.30	0.10	0.13	0.10	0.16	0.10	0.10
	14	0.09	0.09	0.23	0.22	0.10	0.11	0.10	0.19	0.14	0.14
	15	0.13	0.10	0.21	0.24	0.13	0.13	0.13	0.13	0.06	0.07
	16	0.07	0.13	0.26	0.26	0.12	0.10	0.12	0.15	0.12	0.12
	17	0.05	0.06	0.16	0.21	0.10	0.11	0.10	0.18	0.10	0.13
	18	0.13	0.16	0.17	0.24	0.09	0.12	0.09	0.15	0.06	0.09
	19	0.10	0.10	0.17	0.20	0.10	0.09	0.10	0.15	0.09	0.11
	20	0.07	0.09	0.17	0.20	0.05	0.08	0.05	0.17	0.11	0.10
	21	0.05	0.09	0.18	0.23	0.07	0.11	0.07	0.19	0.12	0.12
	22	0.21	0.17	0.28	0.30	0.11	0.12	0.11	0.23	0.11	0.15
**Older Adults**	1	0.12	0.07	0.28	0.28	0.08	0.08	0.19	0.16	0.14	0.11
	2	0.12	0.10	0.24	0.25	0.14	0.14	0.16	0.20	0.11	0.11
	3	0.15	0.13	0.32	0.31	0.11	0.10	0.21	0.19	0.13	0.13
	4	0.14	0.10	0.30	0.28	0.09	0.12	0.20	0.17	0.15	0.13
	5	0.20	0.18	0.32	0.33	0.13	0.12	0.22	0.22	0.16	0.15
	6	0.15	0.14	0.23	0.23	0.14	0.13	0.21	0.20	0.16	0.16
	7	0.10	0.10	0.24	0.22	0.11	0.11	0.18	0.17	0.14	0.13
	8	0.12	0.13	0.25	0.25	0.12	0.10	0.20	0.20	0.14	0.14
	9	0.16	0.11	0.25	0.28	0.11	0.09	0.20	0.18	0.15	0.12
	10	0.14	0.14	0.20	0.22	0.12	0.14	0.20	0.21	0.16	0.16
	11	0.13	0.12	0.26	0.26	0.10	0.12	0.17	0.17	0.14	0.14
	12	0.14	0.12	0.22	0.25	0.12	0.13	0.21	0.21	0.15	0.14
	13	0.12	0.11	0.26	0.27	0.09	0.10	0.16	0.17	0.12	0.12
	14	0.07	0.07	0.26	0.27	0.07	0.08	0.10	0.11	0.09	0.08
	15	0.11	0.11	0.19	0.23	0.10	0.12	0.16	0.16	0.13	0.13
	16	0.14	0.15	0.23	0.23	0.11	0.11	0.18	0.20	0.13	0.13
	17	0.09	0.10	0.19	0.18	0.08	0.11	0.18	0.18	0.13	0.14
	18	0.11	0.10	0.21	0.23	0.09	0.11	0.18	0.18	0.13	0.13

19	0.08	0.09	0.25	0.22	0.15	0.15	0.26	0.27	0.11	0.11
20	0.16	0.16	0.26	0.26	0.11	0.11	0.20	0.19	0.14	0.15
21	0.13	0.11	0.22	0.24	0.10	0.12	0.20	0.20	0.13	0.13
22	0.11	0.10	0.25	0.26	0.12	0.13	0.22	0.20	0.15	0.14
24	0.10	0.07	0.26	0.26	0.11	0.10	0.17	0.18	0.13	0.12
25	0.12	0.11	0.22	0.22	0.09	0.10	0.16	0.16	0.12	0.12
26	0.14	0.15	0.28	0.28	0.13	0.13	0.21	0.22	0.16	0.16
27	0.09	0.09	0.22	0.25	0.08	0.07	0.16	0.16	0.11	0.10

Table 2. Mean (SD) demographics broken down by high and low physical activity levels for healthy older adults and stroke. Hem = hemisphere; mo = month; TSS = time since stroke

	**Older Adults**		**Stroke**	
	High (n = 14)	Low (n = 12)	High (n = 8)	Low (n = 14)
**Sex**	10 F / 4 M	6 F / 6 M	2 F / 6 M	5 F / 9 M
**Age**	62.1 (8.2)	67.3 (8.3)	67.3 (13.9)	64.9 (9.3)
**Stroke Hem**			3 L / 5 R	8 L / 6 R
**TSS (mo)**			43.9 (38.5)	73.3 (49.5)
**Fugl-Meyer**			53.0 (14.8)	44.6 (15.4)
